# Whole-Genome Sequence of *Vibrio alginolyticus* Isolated from the Mucus of the Coral *Fungia danai* in the Andaman Sea, India

**DOI:** 10.1128/genomeA.00339-16

**Published:** 2016-05-05

**Authors:** Tilothama Bhotra, Durg V. Singh

**Affiliations:** Infectious Disease Biology, Institute of Life Sciences, Bhubaneswar, India

## Abstract

*Vibrio alginolyticus*, a halophilic Gram-negative bacterium, which is found in temperate marine and estuarine environments, is known to cause infections in humans and other organisms. We sequenced the genome of sulfamethoxazole-trimthoprim-positive *V. alginolyticus* strain 4-19 isolated from the mucus of the coral *Fungia danai* in the Andaman Sea, India.

## GENOME ANNOUNCEMENT

*Vibrio alginolyticus*, a halophilic Gram-negative bacterium, which is found in temperate marine and estuarine environments, is known to cause human infection (1). This organism also causes infections in corals (2) and in other aquatic organisms, like fish, shrimps, shellfish, and echinoids. Primarily, bacterial strains isolated from corals in the Andaman and Nicobar Islands, India, were tested for the presence of sulfamethoxazole-trimthoprim-related integrating conjugative elements (ICEs) by colony blot assay and *int*SXT PCR (3). Out of five coral samples, three isolates, two from sample 4 and one from sample 5, gave a positive result for the *int*SXT gene and were designated ANC4-1, ANC4-19, and ANC5-1, respectively. The construction of phylogenetic trees using sequences of 16S rRNA and the housekeeping genes *ftsZ*, *gapA*, *gyrB*, *mreB*, *pyrH*, *recA,* and *topA* confirmed the identity of the isolates as belonging to *V. alginolyticus*.

The keys to the evolution of bacteria are mutations, rearrangements, and horizontal gene transfer (HGT), which cause diversification and adaptation of microorganisms (4). ICEs have the combined features of a plasmid and bacteriophage, representing a type of self-transmissible mobile genetic element encoding resistance to multiple antibiotics. It can transfer via conjugation, like a conjugative plasmid, integrate site-specifically, and replicate with the host chromosome, as do many temperate bacteriophages (5).

All three isolates of *V. alginolyticus* showed intermediate resistance to nalidixic acid, and two isolates, ANC4-1, and ANC5-1, showed additional resistance to ampicillin. These isolates were sensitive to other antibiotics tested. All the isolates showed the presence of *tlh* and amplification of a 770-bp fragment. Pulsed-field gel electrophoresis (PFGE) showed a distinct banding pattern among them, indicating diversity in their genomic contents. This study reports the whole-genome sequence of the isolate *V. alginolyticus* 4-19, which will provide information about ICEs in terms of antibiotic resistance, virulence, nitric oxide transcription regulator, 4-carboxymuconolactone decarboxylase, lactate utilization system, ATP-dependent protease, and transposons.

Genomic DNA from *V. alginolyticus* was extracted by a phenol-chloroform extraction method. Whole-genome sequencing was performed using 454 Roche and Illumina sequencing platforms at NxGenBio Life Sciences, Delhi, India. The reads obtained were quality filtered using the FastQC toolkit. The sequence data of the isolate was *de novo* assembled with the help of the Newbler *de novo* assembler. The resulting assemblies generated 139 contigs representing the genome of *V. alginolyticus* strain 4-19, with an average sequencing coverage of 288×. The *de novo*-assembled sequence was further annotated by submitting the sequence to the Rapid Annotations using Subsystems Technology (RAST) server (6). Functional annotation was also performed by PGAAP using the database of the National Center for Biotechnology Information (NCBI).

The chromosome size of the isolate *V. alginolyticus* 4-19 is 5.4 Mb. The G+C content of the strain is 44.4%. After annotation, it was evident that isolate 4-19 contained 2,442 protein-coding genes, 23 RNAs, 63 tRNAs, and 33 rRNAs. The genome analysis showed a relatively high number of coding sequences (CDSs) for the amino acid, carbohydrate, protein, DNA, RNA, and miscellaneous metabolisms in this isolate. The availability of this sequence will facilitate the genomic comparison and understanding of the evolution of ICEs.

### Nucleotide sequence accession number.

The genome sequence of *V. alginolyticus* strain 4-19 has been deposited in DDBJ/EMBL/GenBank under the accession no. LTYK00000000. The version described in this paper is the first version.
